# COVID-19 Chest Manifestation on CT Scan and Associated Risk Factors for Developing Pulmonary Fibrosis

**DOI:** 10.7759/cureus.56616

**Published:** 2024-03-21

**Authors:** Noha Bakhsh, Mai Banjar

**Affiliations:** 1 Department of Medicine, Division of Radiology, Faculty of Medicine in Rabigh, King Abdulaziz University, Jeddah, SAU; 2 Department of Medical Imaging, King Abdullah Medical Complex, Jeddah, SAU

**Keywords:** comorbidities, pulmonary fibrosis, risk factor, computed tomography (ct), covid-19

## Abstract

Purpose: This retrospective study describes the imaging findings on chest computed tomography (CT) scans of coronavirus disease 2019 (COVID-19) patients as well as the prevalence of pulmonary fibrosis and the potential risk factors for the disease.

Methods: One of the major COVID-19 centers in the western province of Saudi Arabia, the King Abdullah Medical Complex in Jeddah, was the site of this study. All adult COVID-19 patients who got a CT chest scan between January 2020 and April 2022 were included in the trial. The imaging findings and pulmonary severity scores (PSS) were obtained from the patients' CT chest. Patients were divided into two groups according to the evidence of fibrotic-like lung changes; clinical and radiological data between the two groups were subsequently compared. Data from the patients' electronic records was collected.

Results: The average patient age was 56.4 years, and most (73.5%) patients were men. Two-thirds of the patients had comorbidities (69.1%). CT scans revealed that diffuse lung infiltration is reported in 61% of cases, followed by lower lobes in 19.9%. Ground glass opacity (94.1%), consolidation (76.5%), septal thickening, and/or reticulation (24.4%) were the main chest findings during the initial CT scan. Fibrotic-like lung changes were developed in 9.6% of patients. Patients known to have a positive history of hypertension (p-value = 0.031) and coronary artery disease (CAD) (p-value = 0.011) were found to be significantly more likely to develop lung fibrosis. The patients’ pneumonia severity score was significantly higher among the lung fibrotic patients (p-value = 0.026). Also, patients who were diagnosed with pulmonary fibrosis stayed longer in the hospital (p-value 0.001). Sex and age did not correlate significantly with risk of lung fibrosis.

Conclusion: Pulmonary fibrosis was observed in 9.6% of COVID-19 patients. A close follow-up of patients with severe pneumonia, prolonged hospitalization, and pre-existing CAD and hypertension was necessary, as pulmonary fibrosis was more likely to occur as a result of these factors. There is a need for a thorough, long-term investigation with a large sample size.

## Introduction

On December 31, 2019, the World Health Organization (WHO) became aware of cases from Wuhan City, China, suffering from respiratory illnesses of unknown origin. The situation progressed and was declared a pandemic on March 12, 2020 [1].

The standard method of diagnosing coronavirus disease 2019 (COVID-19) is real-time reverse-transcription polymerase chain reaction (RT-PCR) [2]. But according to recent research, RT-PCR is only 60-71% sensitive for aiding in the detection of COVID-19 [3,4]. On the other hand, chest CT has been demonstrated to be sensitive to COVID-19 at the time of initial presentation, with a range of 56-98% [3,4].

Acute respiratory distress syndrome (ARDS) can complicate 14% of COVID-19 cases; however, the majority of COVID-19-infected patients exhibit only very mild symptoms, such as a dry cough, fever, and loss of taste or smell [5]. Although COVID-19 is a systemic disease because it can impact multiple organs, it is primarily a respiratory condition [6].

Even though it has been stated that the majority of COVID-19 cases will fully recover after the infection, a number of recent studies suggest that roughly 70-80% of patients will continue to experience a variety of short- or long-term post-infectious complications, especially in severe COVID-19 cases [7,8]. The more severe and often described COVID-19 consequence is pulmonary fibrosis, with the precise incidence of this sequela among COVID-19 survivors and its connection to the viral disease yet to be fully determined [9].

Although by early 2022 the pandemic was controlled in most countries and precautionary measures were lifted, we believe it is necessary to study, understand, document, and compare data from COVID-19 patients in our region with those published in different countries and regions. This study describes the chest findings on CT scans of COVID-19 patients as well as the prevalence of pulmonary fibrosis and the potential risk factors for the disease.

## Materials and methods

This was a retrospective study conducted in a major COVID-19 center, the King Abdullah Medical Complex in Jeddah, in the western province of Saudi Arabia. The study comprised all adult COVID-19 patients who underwent a CT chest scan between January 2020 and April 2022. Patients who had a CT chest scan either at the initial presentation and/or at follow-up were included in this study. Patients who did not have RT-PCR done or who were less than 18 years old were excluded. A total of 136 patients were finally included in the current study. The study was approved by the Institute Review Board of the Ministry of Health/Directorate of Health Affairs in Jeddah (approval number: A01463).

Chest CT protocol

All the patients were imaged with a multi-detector HiSpeed Dual CT scanner (GE HealthCare Technologies, Inc., Chicago, Illinois, United States) using the following parameters: 120 kVp, 150 mA, 0.625-5 mm collimation, 0.9 pitch, and image reconstruction with slice thickness, 2.5 mm/interval. All scans were from the upper thoracic inlet to the inferior costophrenic angle. The patient was supine and at full inspiration during scans. We reviewed the CT chest available at initial and/or follow-up visits for each patient.

Image evaluation

Two radiologists, each with 10 years of experience, reviewed the chest CTs of the COVID-19 patients. The initial and follow-up CT chest images were assessed and compared for the presence of the following features: ground glass opacity, consolidations, septal thickening and/or reticulation, linear bands, thickening of the bronchial wall, nodules, bronchiectasis, pulmonary embolism, and pneumomediastinum, among others. A predominant pattern of chest findings distribution and lung lobe involvement was documented. The PSS and classification were obtained, as described by Chung et al. [1]. The PSS was computed using CT chest scans by adding the scores from each of the five lobes, with each lobe's score being determined independently. PSS ranges from 0 to 20 and a score from 0 to 4 was assigned to each lobe. The extrapulmonary findings and complications of COVID-19 were also recorded.

Patients were divided into two groups according to the evidence of fibrotic-like lung changes on their CT images: (i) the fibrosis group and (ii) the non-fibrosis group. Pulmonary fibrosis on chest CT imaging was defined as a combination of findings, including parenchymal bands, irregular septal thickening, reticulation, and traction bronchiectasis [10]. Clinical and radiological data between the two groups were subsequently compared.

Clinical examination

Patient data including age, gender, nationality, and comorbidities, including diabetes, hypertension, CAD, chronic renal disease, chronic lung disease, and obesity were obtained from the electronic medical records. Data on hospital stays, ICU admissions, and mortalities was also collected. The presenting signs and symptoms of COVID-19 patients were also recorded.

Statistical analysis

The mean and standard deviation were used to describe continuous measured variables, and the metric variables that showed statistical normality assumption violations were described with medians and the interquartile range (IQR). The categorically measured variables were described using percentages and frequencies. The multiple response dichotomy analysis was used to describe the variables with more than one option, like the patients' main CT scan findings. The relationship between categorically measured variables was evaluated using the Chi-squared test of independence. The non-parametric Mann-Whitney U test was used to assess the statistical significance of metric variable differences across the levels of binary and categorically measured factors and variables. The multivariable logistic binary regression analysis was used to assess the statistically significant predictors of the patients' odds of lung fibrosis at admission and follow-up CT scan findings. The association between the predictor variables in the logistic regression analysis was expressed as a multivariate-adjusted odds ratio (OR) with its associated 95% confidence intervals (CIs). The IBM SPSS Statistics for Windows, Version 21.0 (Released 2012; IBM Corp., Armonk, New York, United States) was used for the statistical data analysis, and the alpha significance level was considered at the 0.05 level. 

## Results

Out of 136 patients, 36 (26.5%) were females, and most of them (n=100, 73.5%) were males. The mean age was 56.40 ± 14.30 years. Two-thirds of the patients had comorbidities (n=94, 69.1%) such as diabetes (DM), hypertension, CAD, chronic renal failure, chronic lung disease, and obesity. Other sociodemographic information is given in Table 1. The majority of the patients (64%) required admission to the intensive care units (ICU) upon presenting to the hospital, and of those, 30.1% were deceased. The median hospital length of stay (HLOS) was 21 days (Table 1).

**Table 1:** Sociodemographic and clinical characteristics Data given as frequency (percentage) except in age and hospital length of stay, which are given in mean (SD) and median (IQR), respectively. IQR: interquartile range

Characteristics	Frequency	Percentage
Sex		
Female	36	26.5
Male	100	73.5
Age (years), mean (SD)		56.40 (14.30)
Age group		
20-35 years	14	10.3
36-45 years	19	14
46-55 years	25	18.4
≥56 years	78	57.4
Nationality		
Saudi	103	75.7
Non-Saudi	33	24.3
Comorbidity		
No	42	30.9
Yes	94	69.1
Type of comorbidity		
Hypertension	56	59.6
Diabetes mellitus	66	70.2
Coronary artery disease	17	18.1
Chronic renal failure	7	7.4
Chronic lung disease	9	9.6
Obesity	9	9.6
Others	13	13.8
Length of hospital stay (days), median (IQR)		21 (21)
ICU admission		
No	49	36
Yes	87	64
Mortality		
Yes	41	30.1
No	95	69.9

Shortness of breath was the most common presenting symptom (n=101, 51%), followed by cough (n=39, 19.8%) and fever (n=24, 12.2%), and less frequently other symptoms like nausea and vomiting (n=13, 6.6%), chest pain (n=9, 4.6%), palpitations (n=7, 3.6%), generalized weakness and body aches (n=4, 2%) (Table 2). The PSS was measured in the initial CT, with a median score of 8.78. According to the PSS classification, (n=39, 28.6%) of patients had no or minimal pneumonia, (n=47, 34.6%) had mild pneumonia, (n=44, 32.4%) had moderate pneumonia, and (n=6, 4.4%) of them had severe pneumonia (Table 2). CT scans revealed that peripheral (n=59, 43.4%) and bilateral (n=37, 27.2%) lung involvement were the predominant patterns of distribution. Diffuse lung infiltration is reported in (n=83, 61%) of cases, followed by lower lobes in (n=27, 19.9%) (Table 2).

**Table 2:** Presenting symptoms and radiological characteristics of the patients included in the study Data presented as frequency and percentage except in Pneumonia Severity Score, which is given in median (interquartile range (IQR))

	Frequency	Percentage
Main presenting symptoms		
Shortness of breath	101	51
Cough	39	19.8
Fever	24	12.2
Chest pain	9	4.6
Palpitations	7	3.6
Body aches	4	2
Others	13	6.6
Pneumonia severity score, median (IQR)		8.78 (7.75)
Pneumonia severity score classification		
No or Minimal pneumonia	39	28.6
Mild Pneumonia	47	34.6
Moderate Pneumonia	44	32.4
Severe Pneumonia	6	4.4
Predominant chest findings distribution		
Normal	17	12.5
Bilateral	37	27.2
Central	11	8.1
Peripheral	59	43.4
Unilateral	12	8.8
Predominant lung lobe involvement		
Normal	17	12.5
Diffuse	83	61
Lower Lobe	27	19.9
Middle lobe	2	1.5
Upper lobe	7	5.1

Ground glass opacity (n=112, 94.1%), consolidation (n=91, 76.5%), septal thickening and/or reticulation (n=29, 24.4%), bronchial wall thickening (n=11, 9.2%), and bronchiectasis (n=11, 9.2%) were the main chest findings during the initial CT scan (Table 3). While the most frequent main chest findings during the follow-up exam were ground glass opacity (n=33, 70.2%), septal thickness and/or reticulation (n=18, 38.3%), consolidation (n=14, 29.8%), and bronchiectasis (n=6, 12.8%) (Table 3). The median time from the onset of symptoms to a follow-up CT scan was 60 days (Table 3).

**Table 3:** CT scan findings at initial presentation and follow-up. Time from onset of symptoms to follow-up CT was 60 days.

	Initial CT, n (%)	Follow-up CT, n (%)
Main CT scan findings		
Ground glass opacity	112 (94.1%)	33 (70.2%)
Consolidation	91 (76.5%)	14 (29.8%)
Bronchial wall thickening	11 (9.2%)	0
Nodules	8 (6.7%)	0
Linear bands	0	8 (17%)
Septal thickening AND/OR reticulation	29 (24.4%)	18 (38.3%)
Traction bronchiectasis	11 (9.2%)	6 (12.8%)
Pleural effusion	10 (8.4%)	2 (4.3%)
Pulmonary embolism	4 (3.4%)	0
Atelectasis	2 (1.7%)	2 (4.3%)
Pneumomediastinum	6 (5%)	0
Emphysema	6 (5%)	4 (8.5%)
Empyema	1 (0.8%)	0
Pneumothorax/Hemothorax	8 (6.7%)	2 (4.3%)
Others	10 (8.4%)	9 (19.1%)

Fibrotic-like lung changes were developed in 13 out of 136 patients (9.6%). Sex and age did not correlate significantly with the risk of lung fibrosis. However, patients aged ≥ 56 years were found to be significantly more likely to develop lung fibrosis compared to people in other age groups (p-value = 0.013). Patients known to have a positive history of hypertension and CAD were found to be significantly more likely to develop lung fibrosis compared to those not known to have hypertension or CAD (p-value = 0.031 and p-value = 0.011, respectively). PSS was significantly higher among the lung fibrotic patients (median = 13) compared to those who had no lung fibrotic changes (median score = 10) (p-value = 0.026). The patients who were diagnosed with lung fibrosis stayed longer in the hospital (median HLOS = 44 days) compared to those patients who had no lung fibrosis (median HLOS = 18 days) (p-value = 0.001) (Table 4).

**Table 4:** Correlation between COVID-19 patients’ characteristics and presence of lung fibrosis Data given as frequency (percentage) except in age and hospital length of stay, which are given in mean (SD) and median (IQR), respectively. COVID-19: coronavirus disease 2019; IQR: interquartile range

Characteristics	No Lung Fibrosis (N=123), n (%)	Lung Fibrosis (N=13), n (%)	Test statistic	P-value
Sex				
Female	34 (27.6%)	2 (15.4%)	χ2(1) =0.387	0.534
Male	89 (72.4%)	11 (84.6%)		
Age (years), mean (SD)	55.99 (14.35)	60.23 (13.66)	z (136) =1.57	0.116
Age group				
20-35 years	12 (9.8%)	2 (15.4%)	χ2(3) =10.89	0.013
36-45 years	19 (15.4%)	0		
46-55 years	25 (20.3%)	0		
≥56 years	67 (54.5%)	11 (84.6%)		
Nationality				
Saudi	91 (74%)	92.3%)	χ2(1) =1.27	0.26
Non-Saudi	31 (26%)	1 (7.7%)		
Comorbidity				
No	41 (33.3%)	1 (7.7%)	χ2(1) =2.52	0.112
Yes	82 (66.7%)	12 (93.3%)		
Type of comorbidity				
Hypertension	47 (38.2%)	9 (69.2%)	χ2(1) =4.67	0.031
Diabetes mellitus	59 (48%)	7 (53.8%)	χ2(1) =0.17	0.687
Coronary artery disease	12 (9.8%)	5 (38.5%)	χ2(1) =6.43	0.011
Chronic Renal Failure	7 (5.7%)	0	χ2(1) =0.050	0.823
Chronic Liver Failure/disease	7 (5.7%)	2 (15.4%)	χ2(1) =0.563	0.453
Obesity	9 (7.3%)	0	χ2(1) =0.179	0.673
Others	11 (8.9%)	2 (15.4%)	χ2(1) =0.10	0.799
Presenting sign and symptoms				
Shortness of Breath (SOB)	83 (67.5%)	11 (84.6%)	χ2(1) =0.914	0.339
Cough	32 (26%)	7 (53.8%)	χ2(1) =3.20	0.074
Fever	21 (17.1%)	3 (23.1%)	χ2(1) =0.025	0.875
Nausea & vomiting	11 (8.9%)	2 (15.4%)	χ2 (1) =0.065	0.799
Chest pain	9 (7.3%)	0	χ2(1) =0.18	0.673
Palpitations	7 (5.7%)	0	χ2(1) =0.50	0.823
Hypoxia	4 (3.3%)	1 (7.7%)	χ2(1) =0.001	0.973
Frailty and body aches	4 (3.3%)	0	χ2(1) <0.0001	1
Tachypnea	2 (1.6%)	0	χ2(1) <0.001	1
Pneumonia severity score, median (IQR)	10 (10%)	13 (5%)	Z (136) =2.25	0.026
Pneumonia severity score (PSS) classification				
No or Minimal pneumonia	38 (30.9%)	1 (7.7%)	χ2(4) =10.38	0.035
Mild Pneumonia	42 (34.1%)	5 (38.5%)		
Moderate Pneumonia	40 (32.5%)	4 (30.8%)		
Severe Pneumonia	3 (2.4%)	3 (23.1%)		
Predominant chest findings distribution				
Normal	17 (13.8%)	0	χ2(5) =9.97	0.079
Bilateral	31 (25.2%)	6 (46.2%)		
Central	11 (8.9%)	0		
Peripheral	52 (42.3%)	7 (53.8%)		
Unilateral	12 (9.8%)	0		
Predominant lung lobe involvement				
Normal	17 (13.8%)	0	χ2(6) =6.15	0.407
Diffuse	74 (60.2%)	9 (69.2%)		
Lower lobe	25 (20.3%)	2 (15.4%)		
Middle lobe	2 (1.5%)	0		
Upper lobe	5 (4.1%)	2 (15.4%)		
Main CT scan findings				
Ground glass opacity	100 (81.3%)	12 (92.3%)	χ2(1) =0.40	0.544
Consolidation	85 (69.1%)	6 (46.2%)	χ2(1) =1.85	0.173
Septal thickening AND/OR reticulation	24 (19.5%)	5 (38.5%)	χ2(1) =1.51	0.219
Traction bronchiectasis	4 (3.3%)	7 (53.8%)	χ2(1) =33.96	<0.001
Bronchial wall thickening	8 (6.5%)	3 (23.1%)	χ2(1) =2.40	0.121
Extrapulmonary CT findings				
No	107 (87%)	12 (92.3%)	χ2(1) =0.012	0.912
Yes	16 (13%)	1 (7.7%)		
ICU admission				
No	47 (38.2%)	2 (15.4%)	χ2(1) =1.76	0.185
Yes	76 (61.8)	11 (84.6)		
Length of hospital stay (days), median (IQR)	18 (15)	44 (42.5)	Z (136) =3.56	<0.001
Mortality				
No	87 (70.7%)	8 (61.5%)	χ2(1) =0.14	0.712
Yes	36 (29.3%)	5 (38.5%)		

Regression was applied to the COVID-19 patients’ odds of CT-evidenced lung fibrosis. The patients with a known history of CAD were found to be significantly more inclined (7.17 times more) to have positive evidence of lung fibrotic changes with COVID-19 disease compared to the patients not known to have CAD (p-value = 0.02) (Table 5).

**Table 5:** Multivariable logistic binary regression analysis of odds of CT scan diagnosis with lung fibrosis post COVID-19 disease COVID-19: coronavirus disease 2019

	Multivariate adjusted OR	95% CI		P-value
		Lower	Upper	
Sex (Male)	2.038	.219	18.971	.532
Age (years)	.997	.943	1.053	.907
Pneumonia severity score	1.239	.990	1.551	.061
Coronary artery disease	7.174	1.356	37.948	.020
Constant	.003			.016

## Discussion

The results of the present study are similar to those of the 58 patients in the study by Caruso et al. [11], where consolidation was observed in 42 patients (72%), and ground-glass opacity in 58 patients (100%). Our findings, however, contradict the findings by Zhu et al. [12] and Chung et al. [1], which found a lower prevalence of ground glass opacities. Of the 21 patients in Chung et al., 12 patients (57%) showed ground-glass opacities and six patients (29%) showed consolidation [1]. Of the 31 patients in Zhu et al., only 15 (47%) developed ground glass opacities [12].

Interestingly, bilateral involvement was much lower in the current study (27.2%) versus (76%) in Chung et al. [1] and (91%) in Zhu et al. [12]. Moreover, in prior reports that evaluated CT patterns in COVID-19 patients, there were tendencies for the lung disease to have a basilar and subpleural distribution without a definite lobar or craniocaudal distribution [1,11-13]. Our study showed diffuse and peripheral infiltration to be the most common patterns of involvement.

The primary cause of post-COVID-19 pulmonary fibrosis (PCPF) is still unknown; some theories refer to the abnormal immunological response and the resulting cytokine storm [14]. Previous studies found a wide range of prevalence rates for pulmonary fibrosis in COVID-19 individuals, from 25.5% to 84.15% [15-27]. In our trial, we report a lower prevalence rate that is comparable to Aul et al.'s findings [28].

The literature search revealed similar results regarding the fact that individuals with lung fibrosis were significantly older than those who did not have fibrotic lung changes [11-27]. However, age disparities between those with and without fibrosis were negligible, according to Aul et al. [28]. In the current study, age was found to be irrelevant, although pulmonary fibrosis was significantly more likely to develop in older individuals (≥56 years). As in our analysis, many investigations have not found any correlation between gender and the development of lung fibrosis in COVID-19 cases [16-17,20-26].

A strong association between fibrotic lung disorders and CAD was documented in non-COVID-19 patients [29]. Numerous studies have shown a link between chronic obstructive pulmonary disease (COPD) and PCPF, but no conclusive evidence of an association with CAD has been reported [16-17,23]. According to the current study findings, the most significant comorbidity, which has a strong correlation with PCPF, is CAD. As in earlier investigations, we also discovered a significant association between hypertension and PCPF [16-17,23].

Similar to findings obtained in other studies [22,27,30], we found traction bronchiectasis, septal thickening, and reticulation to be the most frequent CT abnormalities in the fibrotic group, in addition to ground-glass opacity and consolidation. Multiple prior studies, including ours, have identified severity factors for pulmonary fibrosis as a higher pneumonia severity score and a longer hospital stay [15-17,19-26,28].

There are various limitations to the current study. First, the study employed a single-center retrospective design. Second, the sample size was also quite small. Third, a longer follow-up is needed to ascertain if the fibrotic abnormalities were temporary or permanent and whether they cured, stabilized, or worsened with time.

## Conclusions

Pulmonary fibrosis was observed in 9.6% of COVID-19 patients. A close follow-up of patients with severe pneumonia, prolonged hospitalization, and pre-existing CAD and hypertension was necessary, as pulmonary fibrosis was more likely to occur as a result of these factors. There is a need for a thorough, long-term investigation with a large sample size.
